# Women veterans’ experiences of veteran-specific support services: an international scoping review

**DOI:** 10.1186/s12905-026-04512-0

**Published:** 2026-05-02

**Authors:** Lauren Godier-McBard, Claire Hooks, Ellie Buxton, Louise Morgan, Abigail Adams, Matt Fossey

**Affiliations:** 1 Centre for Military Womens’ Research, Veterans and Families Institute for Military Social Research, Faculty of Health, Medicine and Social Care, Anglia Ruskin University, Bishop Hall Lane, Chelmsford, CM1 1SQ United Kingdom; 2 School of Midwifery and Community Health, Faculty of Health, Medicine and Social Care, Bishop Hall Lane, Chelmsford, CM1 1SQ United Kingdom; 3 School of Criminology, Sociology and Social Policy, Loughborough University, Epinal Way, Loughborough, LE11 3TU United Kingdom

**Keywords:** Women, Female, Veterans, Military, Access to care, Barriers, Facilitators, Support services

## Abstract

**Background:**

The growing proportion of women in veteran communities internationally highlights a rising need for veteran support services tailored to their unique experiences. Despite this, support services remain predominantly designed for men, leading to underutilization and dissatisfaction among women veterans. This scoping review aimed to provide a comprehensive international review of the current state of knowledge regarding the experiences of women veterans in accessing and engaging with veteran-specific support services.

**Methods:**

This study followed the Joanna Briggs Institute scoping review methodology. Five databases were searched for papers published from 2000 onwards. Studies reporting on barriers and/or facilitators to access and experiences of engaging with veteran-specific support services reported by women veterans were included. There were no limitations on study methodology or country of origin, and all publications reporting primary research were included.

**Results:**

A total of 117 studies were included in the review. This research originated predominantly from the US (*n* = 109), with seven UK papers, and one Canadian. Eleven themes were identified across the literature, highlighting gendered barriers and facilitators of accessing veteran-specific support for women. Women veterans report feelings of discomfort, exclusion, and discrimination within veteran services, which are perceived as being set up and designed for men. Women report experiencing stigma in help-seeking compounded by a perception of feminine weakness experienced during military service. Some women didn’t want to access services they saw as military-adjacent, due to gendered adverse experiences during military service, including discrimination, harassment, and sexual violence. A lack of identification with the term ‘veteran’ further hinders women’s engagement with veteran-specific services. Enablers of access include care that is sensitive to women’s needs, trauma-informed service user-provider relationships, and peer support.

**Conclusion:**

The reviewed evidence suggests women experience unique challenges and needs in accessing veteran-specific services. Support services should focus on developing care that is, culturally competent, trauma-informed and sensitive to the needs of women, to address gendered barriers to engagement. More research is needed to confirm these research findings outside of the US context, and incorporating an intersectional lens in future research will be essential for improving the support systems for women veterans internationally.

**Supplementary Information:**

The online version contains supplementary material available at 10.1186/s12905-026-04512-0.

## Background

The proportion of women in veteran communities is fast growing, leading to an inevitable increase in their need for, and use of, veterans’ support services [1]. Indeed, in the US, women veterans represent the fastest growing cohort of new users in the Veterans Affairs (VA) healthcare system [2]. Similarly, in the UK, over 1000 women leave the military each year, and the proportion of women in the veteran community is increasing more quickly than anticipated [3, 4]. Despite this, it has been widely argued that support services for veterans have been designed to meet the needs of men, because of their over-representation in the veteran community, and have not yet been adequately adapted to reflect the growing diversity within the veteran population [5].

Veteran research is predominantly focused on veteran men, with one 2019 review reporting that only 2% of all international veteran research mentions women or female veterans [1]. However, the gendered evidence base is growing and indicates that women veterans have unique experiences during and after service, that impacts their support needs and experiences of veteran-specific services.

International research suggests that women are more at risk of experiencing gender-based discrimination, bullying, harassment, and assault during military service, which are associated with poor health and well-being outcomes. Previous research has found that women veterans feel overlooked and undervalued in the men-centric environment of the military [6, 7], and the pressure of trying to fit into the hyper-masculine military culture harms the psychological well-being of servicewomen [8]. Additionally, women are at a higher risk of sexual harassment and sexual assault during military service compared to men [9–11], and these experiences are related to poor mental health outcomes [12]. Considering the differing and gendered experiences of servicewomen, the differing needs of women veterans seeking support post-service must be acknowledged.

Research from the US suggests that women veterans experience less satisfaction with veteran-specific services [13], and underutilise such services in comparison to men [14]. In an examination of gender differences in help-seeking in the UK context, one study found that whilst the proportion of men and women veterans who had accessed mental health support after service was similar, women veterans were significantly more likely to access non-veteran-specific civilian healthcare support compared to men [15]. Further findings from this study suggest that women had experienced barriers to help-seeking including in-service gender discrimination, gender-related stigma, and a lack of understanding from care providers around the issues they face post-service [15]. By underutilising such services women veterans may miss out on tailored support.

There have been several reviews of the literature relating to women veterans’ support needs and access to support in several key areas including: women veterans’ physical health [16, 17], mental health [18, 19], and PTSD [20]. Existing reviews have advanced understanding of discrete health outcomes among women veterans, yet they rarely integrate these within a broader framework that accounts for institutional gender dynamics. Furthermore, few have addressed how veteran-specific service models, which have been developed around the needs of the default veteran man, mediate women’s access, utilisation and satisfaction. This lack of integration has restricted the field’s ability to identify structural and cultural factors that impact support provision for women.

Despite increasing attention to gender in veteran research, previous research remains fragmented. Much of the literature treats women veterans as a comparative subgroup, rather than as a distinct population with specific social, cultural, and institutional experiences. Feminist scholarship has highlighted that much veteran research treats gender primarily as a descriptive variable (for example by “adding” women veterans or comparing women and men) rather than as an analytical category that shapes how veteran status is defined, measured, and experienced [21]. This approach risks obscuring how gender norms and intersectional identities structure women veterans’ experiences. In practice, researchers often use terms such as “women veterans” and “female veterans” interchangeably. Such conceptual ambiguity makes it difficult to distinguish sex‑related from gender‑related processes [22]. In light of these concerns, an important element of this review is to examine how sex and gender are conceptualised and reported within the existing literature on women veterans’ use of veteran‑specific services, alongside mapping substantive findings.

Against this evidential backdrop, there is a need for a comprehensive international synthesis that focuses specifically on women veterans’ experiences of veteran‑specific services and attends to how gender is conceptualised in this literature. This review seeks to: (a) describe how women veterans’ experiences of veteran-specific services have been conceptualised and studied; (b) identify reported barriers and facilitators to accessing and engaging with such services; and (c) highlight gaps in the existing evidence to inform future research, policy, and practice.

## Method

To allow for the inclusion and synthesis of the variety of study designs and methodologies in the relevant research area [23], a scoping review methodology was utilised. The aim of a scoping review is to provide an ‘overview of the existing research base’ [23]. In this instance, to map out the current state of knowledge regarding the experiences of women veterans when using veteran-specific support services. The Joanna Briggs Institute’s updated methodology for undertaking scoping reviews was applied [23, 24]. The review follows PRISMA ScR reporting guidance (see PRISMA checklist in Supplementary Materials).

### Identifying the research question

The overarching research question addressed is: What are the experiences of women veterans in accessing veteran-specific services internationally? Sub-questions included:

1. How have women veterans’ experiences of veteran-specific services been conceptualised and studied?
2. What barriers and facilitators to accessing and engaging with veteran-specific services are reported by women veterans?
3. What gaps in the existing evidence are identified in relation to women veterans’ experience of veteran-specific services?

### Review terminology

For this review ‘veteran-specific’ services included any formal support services which exclusively serve veterans. ‘Statutory’ services refer to services that government and local authorities provide to the public, including civilians and/or veterans. ‘Third sector’ refers to non-government and not-for-profit organisations, including charities, voluntary and community services.

In this review, we use the terms sex and gender in line with contemporary guidance on military and veteran research. Sex refers to biological attributes (e.g. anatomy, chromosomes, hormones), whereas gender refers to socially constructed roles, norms, and identities [22]. Our primary focus is on individuals who identify as women and who have served in the armed forces (hereafter “women veterans”). Where we refer to sex-specific issues (e.g. pregnancy, childbirth, menopause, gynaecological health), we use female to denote biological sex. Where we discuss norms, discrimination, roles, and identities (e.g. gender-based discrimination, gendered stigma, gender-sensitive care), we use gender terminology. Unless otherwise stated, our focus on gendered experiences relates to women veterans; where studies include men or other gender identities, this is explicitly specified. We recognise that existing services are already gendered in practice, having historically centred men as the default veteran, and we therefore use “gender-sensitive for women” when referring to care that explicitly addresses women’s needs. In Table 2, where primary studies use sex/gender terms inconsistently or without definition, we use the authors’ terminology and note this limitation.

### Identifying relevant studies

The review search process was conducted initially in May 2022 and updated in October 2023. We searched the following academic databases for articles published after 2000; Web of Science, PubMed, Scopus, PsychINFO, U.K Parliamentary Papers. Additionally, searches for grey literature were carried out in the Veterans and Families Research Hub, Google Scholar (examining the first 10 pages of search results), GOV.UK, Open Grey, RAND, ProQuest Dissertations and Theses. Reference lists of relevant journals, books, and articles were also hand-searched. Keywords including ‘experiences’, ‘barriers’, ‘facilitators’, ‘support services’ ‘woman’, and ‘veteran’ were used in the search string for each database along with Boolean operators (see Table 1). The composition and structure of these search strings were altered according to the requirements of each database.

**Table 1:** Search terms and inclusion/exclusion criteria

Keyword	Search String
Barriers	Barrier* OR Obstacle* OR Hurdle* OR Imped* OR Difficult*
Facilitators	OR facilitat* OR promot* OR aid* OR Enabl* OR help*
Experiences	OR reason* OR experienc* OR Perception*
Support service	AND charity OR statutory OR government OR service utili*ation OR service use OR service access* OR service* support OR support service* OR care service*
Female gender	AND Women OR woman OR female
Veteran	AND Veteran* OR ex-service OR ex-forces OR ex-military OR ex-soldier*
Inclusion criteria	All methodologies are to be included. Directly investigates women or female military veterans’ experiences of veteran-specific services. Original research with primary data published in peer-reviewed journals, grey literature, and governmental reports are to be included. Published after 2000
Exclusion criteria	Articles not written in English. The article addresses issues other than those detailing women or female military veteran experiences/barriers/facilitators (e.g. papers presenting associations between socio-demographic information and service utilisation or satisfaction). Articles discussing only non-veteran-specific services. Published before 2000

### Study selection

The inclusion criteria for this study required papers to focus on the experiences of women veterans in accessing veteran-specific services and meet the inclusion/exclusion criteria as detailed in Table 1. Initially, titles were screened followed by abstracts, and then a full-text review of relevant texts carried out by two researchers. Following this process, a third researcher was consulted on any papers that were still in question to make the final decision on their inclusion. The reference and citation lists for each included article were also searched to identify any additional papers of relevance. The articles were not quality assessed in accordance with the Joanna Briggs Institute’s updated methodology for undertaking scoping reviews which aim to provide an ‘overview of the existing evidence base regardless of quality’ (pp.143).

### Charting the data

The following data from the included articles was extracted and charted; full journal reference/authors, publication date, service branch/es, country of origin, study population, aim of the study, which statutory/third sector services were included, methodology used, and main findings. Initial data extraction was conducted by one researcher, and a subsample of extractions was independently checked by two other researchers to ensure consistency and accuracy. Discrepancies were resolved through discussion among the three researchers. The gender/sex terminology used by the papers was subsequently extracted and added to the table. We use the original terms used to define samples by the study authors, and identify where sex/gender terms have been used without definition or explanation.

A descriptive reflexive thematic analysis [25, 26] was used to identify common themes relating to veteran’s experiences, including experience with services, and barriers and facilitators to access and engagement. Initial inductive line‑by‑line coding was conducted by one researcher. The researcher then combined similar line-by-line codes to form a preliminary coding framework. Codes were then combined and developed further into themes and subthemes through discussion among three researchers focusing on results relevant to the research question and objectives outlined above. A second researcher coded a subsample of papers to check the stability and consistency of the coding framework.

An overview of the paper’s characteristics and themes identified is provided in Table 2.

**Table 2:** Characteristics of included papers

Authors & Title	Year of publication	Country of origin	Sample & sex/gender terms used	Type of veteran support service	Methodology	Themes
Al Masarweh, L. I. ‘Barriers to Native American Women Veterans’ Health Care Access on Two Reservations: Northern Cheyenne and Flathead’	2014	US	15 Native American women veterans*	Statutory (VHA) and Indian Health Services	Qualitative: Semi-structured interviews	Awareness of services and eligibility Accessibility
Al Masarweh, L & Ward, C. ‘Barriers to health care access and utilization: a study of native American women veterans in two Montana reservations’	2016	US	18 Native American women veterans*	Statutory (VHA) and Indian Health Services	Qualitative: Semi- structured interviews	Awareness of services and eligibility Accessibility Gender-sensitive care for women. Help-seeking stigma Peer support
Arnold, A. ‘Let the Healing Begin: Exploring the Relationship Between Military Trauma and Women Veterans’ Use of Veterans Health Administration Services’	2012	US	595 women veterans*, nationally representative sample	Statutory (VHA)	Quantitative: Cross sectional survey	Awareness of services and eligibility
Baumann, J. Williamson, C. Murphy, D. ‘Exploring the impact of gender-specific challenges during and after military service on female UK Veterans’	2022	UK	750 female veterans* (Army only), members of charitable organisation	Third Sector/charitable and Statutory (NHS)	Quantitative: Cross sectional survey	Discomfort in men-centric environments.
Bean-Mayberry, A. B. Chang, C. H. McNeil, M. A. Whittle, J. Hayes, P. M. Hudson Scholle, S. ‘Patient Satisfaction in Women’s Clinics Versus Traditional Primary Care Clinics in the Veterans Administration’	2003	US	971 female/women veterans**, enrolled in VHA care.	Statutory (VHA)	Quantitative: Cross sectional survey	Discomfort in men-centric environments. Gender-sensitive care for women.
Bradwisch, S. ‘The Experience of Female Veterans’ Transitioning to The Experience of Female Veterans’ Transitioning to Post—Active— Duty.’	2019	US	11 female veterans*, discharged between 1990–2017.	Any veteran services, including statutory (VHA), third sector, community, or private.	Qualitative: Semi-structured interviews	Continuity/integration of services Awareness of services and eligibility Accessibility Gender discrimination against women from service providers
Brooks, E. Dailey, N. K. Bair, B. D. and Shore, J. H. ‘Listening to the Patient: Women Veterans’ Insights About Health Care Needs, Access, and Quality in Rural Areas.’	2016	US	35 women veterans* living in rural areas.	Any veteran services, including statutory (VHA), third sector, community, or private.	Qualitative: Semi-structured interviews	Accessibility Discomfort in men-centric environments. Peer support Gender-sensitive care for women. Negative military experiences
Burnett-Zeigler, I. Zivin, K. Ilgen, M. A. and Bohnert, A. S.B. ‘Perceptions of quality of health care among veterans with psychiatric disorders.’	2011	US	55,578 female and male veterans enrolled in VHA care***, diagnosed with a psychiatric disorder	Statutory (VHA)	Quantitative: Cross sectional survey	Service user-provider relationship
Callegari, L. S. Borrero, S. Reiber, G. E. Nelson, K. M. Zephyrin, L. Sayre, G. G. Katon, J. G. ‘Reproductive Life Planning in Primary Care: A Qualitative Study of Women Veterans’ Perceptions.’	2015	US	27 women veterans* aged 18–44 years, enrolled in VHA care.	Statutory (VHA)	Qualitative: Semi-structured interviews	Service user-provider relationship
Callegari, L. S. Tartaglione, E. V. Magnusson, S. L. Nelson, K. M. Arteburn, D. E. Szarka, J. Zephyrin, L. Borrero, S. ‘Understanding Women Veterans’ Family Planning Counseling Experiences and Preferences to Inform Patient-Centered Care.’	2019	US	32 women veterans* aged 18–44 years, enrolled in VHA care	Statutory (VHA)	Qualitative: Semi-structured interviews	Gender discrimination against women from service providers Service user-provider relationship Continuity/integration of care
Campbell, G. M., & Murphy, D. ‘ENHANCE Study: Improving access to evidence-based treatment for women veteran survivors of sexual trauma.’	2023	UK	19 women veterans, enrolled in mental healthcare from third sector organisation	Third sector organisation	Qualitative: Semi-structured interviews	Help-seeking stigma Accessibility Gender-sensitive care for women. Peer support Discomfort in men-centric environments. Negative military experiences Veteran identity
Campbell, G. M., Williamson, V., & Murphy, D. ‘”A Hidden Community”: The Experiences of Help-Seeking and Receiving Mental Health Treatment in U.K. Women Veterans. A Qualitative Study.’	2023	UK	19 women veterans*, enrolled in mental healthcare from third sector organisation	Third sector organisation	Qualitative: Semi-structured interviews	Help-seeking stigma Accessibility Gender-sensitive care for women. Peer support Discomfort in men-centric environments. Negative military experiences Veteran identity
Carlson, G. C. Than, C. T. Rose, D. Brunner, j. Canfreau-Coffiner, C. Canelo, I. A. Klap, R. Bean-Mayberry, B. Agrawl, (A) Hamilton, A. (B) Gerber, M. R. Yano, E. M. ‘What Drives Women Veterans’ Trust in VA Healthcare Providers?’	2022	US	1395 women veterans*, enrolled in VHA care	Statutory (VHA)	Quantitative: Cross sectional survey	Service user-provider relationship Gender-sensitive care for women.
Carr, S. F. ‘Factors that impact female utilization of Veterans’ Health Administration services.’	2020	US	2340 women veterans*, enrolled in VHA care	Any veteran services, including statutory (VHA), third sector, community, or private.	Quantitative: Cross sectional survey	Accessibility Service-user provider relationship
Chanfreau-Coffinier, C. Washington, D. L. Chuang, E. Brunner, J. Darling, J. E. Canelo, L. Yano, E. M. ‘Exploring the association of care fragmentation and patient ratings of care quality: A mediation analysis of women Veterans’ experience with VA care.’	2019	US	1395 women veterans*, enrolled in VHA care	Statutory (VHA)	Quantitative: Cross sectional survey	Continuity/integration of services Gender-sensitive care for women.
Cichowski, S. Ashley, M. Oritz, O. Dunivan, G. ‘Female Veterans’ Experiences With VHA Treatment for Military Sexual Trauma.’	2019	US	17 female veterans* with experience of MST, enrolled in VHA care	Statutory (VHA)	Qualitative: Focus groups	Discomfort in men-centric environments. Gender discrimination against women from service providers Service user-provider relationship Gender-sensitive care for women. Negative military experiences
Chrystal, J. G. Frayne, S. Dyer, K. E. Moreau, J. L. Gammage, C.E. Saechao, F. Berg, E. Washington, D. L. Yano, E. M. Hamilton, A. B. (2022) ‘ Women Veterans’ Attrition from the VA Health Care System.’	2022	US	51 women veterans* with experience of VHA care	Statutory (VHA)	Qualitative: Semi-structured interviews	Accessibility Gender-sensitive care for women.
Department of Veterans Affairs. ‘Study of Barriers for Women Veterans to VA Health Care’.	2015	US	8532 women veterans*, who had and had not used VHA services	Statutory (VHA)	Quantitative: Cross sectional survey	Awareness of services and eligibility Accessibility Help-seeking stigma Discomfort in men-centric environments. Gender-sensitive care for women.
Dichter, M. E. Wagner, C. Goldberg, E. B. and Iverson, K. M. ‘Intimate partner violence detection and care in the Veterans Health Administration: Patient and provider perspectives.’	2015	US	25 female/women veterans**, enrolled in VHA care & 15 VHA care providers	Statutory (VHA)	Qualitative: Semi-structured interviews	Discomfort in men-centric environments. Service user-provider relationship
Dichter, M.E., Wagner, C. and True, G. ‘Women Veterans’ Experiences of Intimate Partner Violence and Non-Partner Sexual Assault in the Context of Military Service: Implications for Supporting Women’s Health and Well-Being.’	2018	US	25 women veterans* enrolled in VHA care.	Statutory (VHA)	Qualitative: Semi-structured interviews	Help-seeking stigma
Di Leone, B. A. L., Wang, J. M., Kressin, N., & Vogt, D. ‘Women’s Veteran Identity and Utilization of VA Health Services.’	2015	US	407 female/women veterans** who served in Afghanistan and/or Iraq	Statutory (VHA)	Quantitative: Cross sectional survey	Veteran identity
Dognin, J. Sedlander, E. Jay, M. and Ades, V ‘Group education sessions for women veterans who experienced sexual violence: Qualitative findings.’	2017	US	27 women veterans* with experience of sexual violence, enrolled in VHA care.	Statutory (VHA)	Qualitative: Focus groups	Service user-provider relationship Peer support Gender-sensitive care for women.
Driscoll, M. A. Knobf, M. T. Higgins, D. M. Heapy, A. Lee, A. and Haskell, S. ‘Patient experiences navigating chronic pain management in an integrated health care system: A qualitative investigation of women and men.’	2018	US	48 veterans (22 female and 26 male)**, enrolled in VHA care	Statutory (VHA)	Qualitative: Focus groups	Continuity/integration of services Service user-provider relationship Accessibility Discomfort in men-centric environments. Gender discrimination against women from service providers Gender-sensitive care for women.
Dyer, K. E. Potter, S. J. Hamilton, A. B. Luger, T. M. Bergman, A. A. Yano, E. M. Klap, R. ‘Gender Differences in Veterans’ Perceptions of Harassment on Veterans Health Administration Grounds.’	2019	US	95 veterans (38 women, 57 men)*, enrolled in VHA care	Statutory (VHA)	Qualitative: Focus groups	Discomfort in men-centric environments.
Evans, E. (A) Tennenbaum, D. L. Hamilton, A. (B) ‘Why Women Veterans Do Not Use VA-Provided Health and Social Services: Implications for Health Care Design and Delivery.’	2019	US	22 women veterans*	Statutory (VHA)	Qualitative: Semi-structured interviews	Continuity/integration of services Awareness of services and eligibility Accessibility Negative military experiences Service user-provider relationship Discomfort in men-centric environments. Gender-sensitive care for women.
Felder, P. Delany, J. P. ‘The life course of homeless female Veterans: Qualitative study findings.’	2020	US	14 homeless female veterans*	Statutory (VHA)	Qualitative: Semi-structured interviews	Awareness of services and eligibility Continuity/integration of services
Ferras, M. Dye, J. Ayala, G. X. Schmied, E. ‘An Examination of Factors That Influence Receipt of Reproductive Health Screenings Among Female Veterans.’	2023	US	90 female/women** veterans, community sample.	Any veteran services, including statutory (VHA), third sector, community, or private.	Quantitative: Cross sectional survey	Accessibility Gender-sensitive care for women.
Ferry, B.J. ‘Exploring mental health treatment for female veterans in the US: assessing the influences on female veterans in selection of treatment location, comparing VA and non-VA settings.’	2016	US	24 female/women** veterans, enrolled in VHA or non-VHA mental healthcare	Any veteran services, including statutory (VHA), third sector, community, or private.	Mixed methods: Cross sectional quantitative survey, including open qualitative questions	Accessibility Gender-sensitive care for women. Discomfort in men-centric environments. Peer support Service user-provider relationship
Fiedler, J. ‘Barriers for Female Veterans Diagnosed with Combat-related Post-traumatic Stress Disorder: A Case Study’	2019	US	5 female veterans* with a diagnosis of combat-related PTSD, enrolled in VHA care.	Statutory (VHA)	Qualitative: Semi-structured interviews	Help-seeking stigma Service user-provider relationship Gender discrimination against women from service providers Accessibility Gender-sensitive care for women.
Fitzke, R.E., Bouskill, K.E., Sedano, A., Tran, D.D., Saba, S.K., Buch, K., Hummer, J.F., Davis, J.P. and Pedersen, E.R., ‘Barriers and Facilitators to Behavioral Healthcare for Women Veterans: a Mixed-Methods Analysis of the Current Landscape.’	2023	US	83 cisgender women veterans in survey, 18 interviewed	Any veteran services, including statutory (VHA), third sector, community, or private.	Mixed methods: Cross sectional survey and semi-structured interviews	Gender-sensitive care for women. Discomfort in men-centric environments.
Fontana, A. Rosenhech, R. ‘Treatment of Female Veterans with Posttraumatic Stress Disorder: the Role of Comfort in a Predominantly Male Environment’	2006	US	224 female veterans*, enrolled in VHA care	Statutory (VHA)	Quantitative: Longitudinal survey	Discomfort in men-centric environments.
Fox, A. B. Meyer, E. C. and Vogt, D. ‘Attitudes about the VA health-care setting, mental illness, and mental health treatment and their relationship with VA mental health service use among female and male OEF/OIF veterans’	2015	US	278 (164 women veterans* with probable PTSD, enrolled in VHA care	Statutory (VHA)	Quantitative: Cross sectional survey	Help-seeking stigma Awareness of services and eligibility
Friedman, S. (A) Frayne, S. M. Berg, E. Hamilton, A. (B) Washington, D. L. Saechao, F. Maisel, N. (C) Lin, J. Y. Hoggatt, K. J. and Phibbs, C. S. ‘Travel time and attrition from VHA care among women veterans: How far is too far?’	2015	US	266,301 women veterans*, enrolled in VHA outpatient care	Statutory (VHA)	Quantitative: Cross sectional survey	Accessibility
Galloway-Salazar, Q. A. ‘Post-9/11 Women Veterans’ Experiences Transitioning Into the Civilian Workforce’	2021	US	8 women veterans* who served post 9/11	Statutory (VHA) and third sector organisations	Qualitative: Semi-structured interviews	Peer support Discomfort in men-centric environments. Accessibility Gender discrimination against women from service providers
Gayles, A. ‘Understanding the Mental Healthcare Needs of Female Veterans and Access to Mental Health Care Services within a Veterans Health Care Facility: A Phenomenological Case Study’	2021	US	4 female veterans* enrolled in VHA care	Statutory (VHA)	Qualitative: Semi-structured interviews	Discomfort in men-centric environments. Service user-provider relationship Gender-sensitive care for women. Continuity/integration of services Accessibility
Gawron, L., Pettey, B. P. W. Redd, A. Suo, Y. Turok, K. D. Gundlapalli, V. A. ‘The “Safety Net” of Community Care: Leveraging GIS to Identify Geographic Access Barriers to Texas Family Planning Clinics for Homeless Women Veterans’	2017	US	3246 homeless women veterans*	Statutory (VHA)	Quantitative: Cross sectional survey	Accessibility
Godier-McBard, L., Cable, G., Wood, A., & Fossey. ‘Gender differences in barriers to mental healthcare for UK military veterans: a preliminary investigation.’	2021	UK	100 veterans (43 female/women**)	Any veteran services, including statutory (NHS), third sector, community, or private.	Mixed methods: Cross sectional survey, with open qualitative questions	Help-seeking stigma Negative military experiences Gender-discrimination against women from service providers
Graham, K., Murphy, D., & Hendrikx, L. J. ‘Exploring Barriers to Mental Health Treatment in the Female Veteran Population: A Qualitative Study’	2023	UK	61 female/women** Army veterans, members of third sector organisation	Any veteran services, including statutory (NHS), third sector, community, or private.	Qualitative: Open survey question	Gender discrimination against women from service providers Help-seeking stigma Accessibility Gender-sensitive care for women. Veteran identity
Hamilton, A. B. Poza, I. Hines, V. Washington, D. L. ‘Barriers to Psychosocial Services among Homeless Women Veterans’	2012	US	29 homeless women veterans*	Statutory (VHA)	Qualitative: Focus groups	Awareness of services and eligibility Gender-sensitive care for women. Gender discrimination against women from service providers Accessibility Continuity/integration of services
Hamilton, B. A. Frayne, M. S. Cordasco, M. K. Washington, L. D. ‘Factors Related to Attrition from VA Healthcare Use: Findings from the National Survey of Women Veterans’	2013	US	2691 women veterans* enrolled or previously enrolled in VHA care	Statutory (VHA)	Quantitative: Cross sectional survey	Gender-sensitive care for women. Accessibility
Hoffmire, C. A. Brenner, L. A. Katon, J.Gaeddert, L. A. Miller, C. N. Schneider, A. L. Monteith, L. L. ‘Women Veterans’ Perspectives on Suicide Prevention in Reproductive Health Care Settings: An Acceptable, Desired, Unmet Opportunity’	2022	US	21 women veterans*, enrolled in VHA reproductive health care	Statutory (VHA)	Qualitative: Semi-structured interviews	Service user-provider relationship Gender-sensitive care for women. Accessibility Help-seeking stigma
Hooks, C., Morgan, L., Fossey, M., Buxton, E., & Godier-McBard, L. ‘Where are all the women?’ Recognition and representation – UK female veterans’ experiences of support in civilian life.’	2023	UK	85 female veterans*	Any veteran services, including statutory (NHS), third sector, community, or private	Qualitative: Semi- structured interviews	Awareness of services and eligibility Discomfort in men-centric environments. Help-seeking stigma Accessibility Gender-sensitive care for women. Peer support Veteran identity
Hundt, E. N. Robinon, A. Arney, J. Stanley, A. M. Cully, A. J. ‘Veterans’ Perspectives on Benefits and Drawbacks of Peer Support for Posttraumatic Stress Disorder’	2015	US	6 female veterans and 17 male veterans**, enrolled in VHA care for PTSD.	Statutory (VHA)	Qualitative: Semi-structured interviews	Peer support Gender-sensitive care for women.
Ingelse, K. Messecar, D. ‘Rural Women Veterans’ Use and Perception of Mental Health’	2016	US	10 rural female/women** veterans, enrolled in VHA care.	Statutory (VHA)	Qualitative: Semi-structured interviews	Peer support Veteran identity Help-seeking stigma Gender discrimination against women from service providers
Jacobs, C. ‘Betrayal by Comrades-in-arms: VA Health Care Experiences of Women Survivors of Military Sexual Trauma’	2016	US	16 women veterans, with experience of MST, enrolled in VHA care	Statutory (VHA)	Qualitative: Semi-structured interviews	Negative military experiences Discomfort in men-centric environments. Gender discrimination against women from service providers Gender-sensitive care for women. Service user-provider relationship Accessibility Continuity/integration of care Peer support
Katon, J. G. Erica W. Sayre, G. Zephyrin, L. C. Cordasco, K. M. Yano, E. M. and Fortney, J. C. ‘Women veterans’ experiences with Department of Veterans Affairs maternity care: Current successes and targets for improvement’	2018	US	27 women veterans*, enrolled in VHA maternity care.	Statutory (VHA)	Qualitative: Semi-structured interviews	Accessibility Service user-provider relationship Gender-sensitive care for women.
Kimerling, R. Pavao, J. Valdez, C. Mark, H. Hyun K. J. Saweikis, M. ‘Military Sexual Trauma and Patient Perceptions of Veteran Health Administration Health Care Quality’	2011	US	164,632 veterans enrolled in VHA outpatient care (5758 women veterans*)	Statutory (VHA)	Quantitative: Cross-sectional survey	Accessibility Service user-provider relationship Negative military experiences
Kimerling, R. Pavao, J. Greene, L. Karpenko, J. Rodriguez, A. Saweikis, M. and Washington, D. L. ‘Access to mental health care among women Veterans: is VA meeting women’s needs?’	2015	US	6287 women veterans*, enrolled in VHA care.	Statutory (VHA)	Quantitative: Cross sectional survey	Gender-sensitive care for women. Accessibility
King, P. R., Buchholz, L. J., Tauriello, S., & Wray, L. O. ‘Qualitative exploration of factors influencing women veterans’ disordered eating symptoms and treatment preferences in VHA primary care.’	2023	US	12 women veterans* who report disordered eating, enrolled in VHA care	Statutory (VHA)	Qualitative: Semi-structured interviews	Service user-provider relationship Help-seeking stigma Accessibility
Kirby, L. ‘Veterans’ Perceptions of Behavioral Health Services for Posttraumatic Stress’	2020	US	8 veterans (5 female and 3 male**), who had served in the Iraq or Afghanistan conflicts	Any veteran services, including statutory (VHA), third sector, community, or private.	Qualitative: Semi-structured interviews	Gender-sensitive care for women.
Kehle-Forbes, S.M. Harwood, E.M. Spoont, M.R. Sayer N.A. Gerould, H. Murdoch, M. ‘Experiences with VHA care: a qualitative study of US women veterans with self-reported trauma histories’	2017	US	37 women* veterans, Vietnam and post-Vietnam era	Statutory (VHA)	Qualitative: Semi-structured interviews	Discomfort in men-centric environments. Gender discrimination against women from service providers
Kelly, M. M. Vogt, S. D. Scheiderer, M. E. Ouimette, P. Daley, J. Wolfe, J. ‘Effects of Military Trauma Exposure on Women Veterans’ Use and Perceptions of Veterans Health Administration Care’	2008	US	1496 women veterans*, national sample	Statutory (VHA)	Quantitative: Cross sectional survey	Negative military experiences
Kelly, U. A. ‘Barriers to PTSD treatment-seeking by women veterans who experienced military sexual trauma decades ago: The role of institutional betrayal’	2021	US	14 women veterans* within experience of MST, enrolled in VHA care	Statutory (VHA)	Qualitative: Semi-structured interviews	Discomfort in men-centric environments. Negative military experiences Gender-sensitive care for women.
Klap, R. Darling, J. E. Hamilton, A. B. Rose, D. E. Dyer, K. Canelo, I. Haskell, S. and Yano, E. M. ‘Prevalence of stranger harassment of women veterans at Veterans Affairs medical centers and impacts on delayed and missed care’	2019	US	7708 female/women** veterans, enrolled in VHA care	Statutory (VHA)	Quantitative: Telephone survey	Discomfort in men-centric environments.
Koblinsky, S. A. Schroeder, A. L. & Leslie, L. A. “Give us respect, support and understanding”: Women veterans of Iraq and Afghanistan recommend strategies for improving their mental health care’	2017	US	29 women veterans*, who served in the Iraq and/or Afghanistan conflicts	Statutory (VHA) and community services	Qualitative: Semi structured interviews	Veteran identity Help-seeking stigma Service user-provider relationship Discomfort in men-centric environments. Gender-sensitive care for women. Awareness of services and eligibility Accessibility Peer support
Kroll-Desrosiers, A. Crawford, S. L.; Moore S. Tiffany A. Clark, M. A. and Mattocks, K. M. ‘Treatment and management of depression symptoms in pregnant veterans: Varying experiences of mental health care in the prenatal period’	2020	US	142 pregnant women veterans*, enrolled in VHA care	Statutory (VHA)	Mixed methods: Cross sectional survey & semi-structured interviews	Continuity/integration of care Accessibility Service user-provider relationship
Kroll-Desrosiers, A. Kinney, R. L. Marteeny, V. Mattocks, K. M. ‘Exploring the Acceptability of Expanded Perinatal Depression Care Practices Among Women Veterans’	2022	US	27 pregnant and postpartum women veterans*	Statutory (VHA)	Qualitative: Semi-structured interviews	Gender-sensitive care for women. Peer support
Lavrov, B. A Nethery, S. A. ‘Women who have served in the military: subjective meanings and self perceptions as a veteran and the impact on access to veterans services’	2005	US	16 women veterans*	Statutory (VHA)	Qualitative: Semi-structured interviews	Gender-sensitive care for women. Awareness of services and eligibility Continuity/integration of services Accessibility Peer support Negative military experiences
Lafferty, M., Winchell, K., Cottrell, E., Knight, S. and Nugent, S.M. ‘Women of the Gulf War: understanding their military and health experiences over 30 years.’	2023	US	10 women veterans* of the Gulf War	Any veteran services, including statutory (VHA), third sector, community, or private.	Qualitative: Semi-structured interviews	Gender-sensitive care for women. Service user-provider relationship Discomfort in men-centric environments.
Lehavot, K. Der-Martirosian, C. Simpson, T. L. Sadler, A. G. and Washington, D. L. ‘Barriers to care for women veterans with posttraumatic stress disorder and depressive symptoms’	2013	US	3593 women veterans* in nationally representative sample, screened for PTSD and depression	Statutory (VHA)	Quantitative: Cross sectional survey	Accessibility Awareness of services and eligibility
Leslie, L. A. & Koblinsky, S. A. ‘Returning to civilian life: Family reintegration challenges and resilience of women veterans of the Iraq and Afghanistan wars.’	2017	US	29 women veterans*	Any veteran services, including statutory (VHA), third sector, community, or private.	Qualitative: Focus groups	Peer support Gender-sensitive care for women.
Lewis, E. T. Jamison, (A) L. Ghaus, S. Durazo, E. M. Frayne, S. M. Hoggatt, K. J. Bean-Mayberry, (B) Timko, (C) & Cucciare, M. A. ‘Receptivity to alcohol-related care among U.S. women Veterans with alcohol misuse’	2016	US	30 women veterans* screened positive for alcohol misuse, enrolled in VHA care	Statutory (VHA)	Qualitative: Semi-structured interviews	Service user-provider relationship Gender-sensitive care for women.
MacDonald, S. Judge-Golden, C. Borrero, S. Zhao, X. H. More, M. K. Hausmann, L. R. M. ‘Experiences of Perceived Gender-based Discrimination Among Women Veterans Data From the ECUUN Study.’	2020	US	2294 women veterans* enrolled in VHA care	Statutory (VHA)	Quantitative: Cross sectional survey	Gender discrimination against women from service providers Gender-sensitive care for women.
Mattocks, K. M. Nikolajski, C. Haskell, S. Brandt, C. McCall-Hosenfeld, J. Yano, E. Pham, T. and Borrero, S. ‘Women veterans’ reproductive health preferences and experiences: a focus group analysis’	2011	US	25 women veterans* enrolled in reproductive care at VHA	Statutory (VHA)	Qualitative: Focus groups	Gender-sensitive care for women. Awareness of services and eligibility Gender discrimination against women from service providers
Mattocks, K. M. Kuzdeba, J. Baldor, R. Casares, J. Lombardini, L. and Gerber, M.R. ‘Implementing and Evaluating a Telephone-Based Centralized Maternity Care Coordination Program for Pregnant Veterans in the Department of Veterans Affairs’	2017	US	103 women veterans* under VHA maternity care	Statutory (VHA)	Mixed methods: Cross sectional survey with closed and open questions	Gender-sensitive care for women.
Mattocks, K.M. Yano, E. M. Brown, A. Casares, J. and Bastian, L. ‘Examining Women Veteran’s Experiences, Perceptions, and Challenges With the Veterans Choice Program’	2018	US	148 women veterans*, enrolled in VHA care	Statutory (VHA)	Qualitative: Semi-structured interviews	Awareness of services and eligibility Accessibility
Mattocks, K. M. Kroll-Desrosiers, A. Kinney, R. and Singer, S. ‘Understanding Maternity Care Coordination for Women Veterans Using an Integrated Care Model Approach’	2019	US	276 postpartum women veterans*, enrolled in VHA maternity services	Statutory (VHA)	Quantitative: Cross-sectional survey	Gender-sensitive care for women.
Mattocks, K. M. Baldor, R. Bean-Mayberry, B. Cucciare, M. Gerber, M. R. Goldstein, K. M. Hammer, K. D. Hill, E. E. Kroll-Desrosiers, A. Prochazka, A. V. Sadler, A. G. Bastian, L. ‘Factors impacting perceived access to early prenatal care among pregnant veterans enrolled in the Department of Veterans Affairs’	2019	US	581 pregnant women veterans*, enrolled in VHA maternity care	Statutory (VHA)	Quantitative: Telephone survey	Accessibility
Mattocks, K. Casares, J. Brown, (A) Bean-Mayberry, (B) Goldstein, K. M. Driscoll, M. Haskell, S. Bastian, L. Brandt, (C) ‘Women Veterans’ Experiences with Perceived Gender Bias in U.S. Department of Veterans Affairs Specialty Care’	2020	US	80 women veterans* who served during the Iraq and Afghanistan conflicts	Statutory (VHA)	Qualitative: semi-structured interviews	Gender discrimination against women from service providers Gender-sensitive care for women.
McBain, S. A. Garneau-Fournier, J. Turchik, J. A. ‘The Relationship Between Provider Gender Preferences and Perceptions of Providers Among Veterans Who Experienced Military Sexual Trauma’	2022	US	1591 veterans (1123 women), with experience of MST, received at least one VHA outpatient service.	Statutory (VHA)	Quantitative: Cross sectional survey	Gender-sensitive care for women.
McDonald, K.L., Meunier, C.C., Liu, B., Roth, B. and Denneson, L.M., “Gender differences in barriers and facilitators to care among US military veterans at high risk for suicide: a qualitative study.”	2023	US	25 women veterans and 25 men veterans using VHA-services, with a recent suicide attempt.	Statutory (VHA)	Qualitative: Semi-structured interviews	Discomfort in men-centric environments. Veteran Identity. Negative military experiences Gender sensitive care for women. Service user-provider relationship Peer support
Mengeling, M. A. Sadler, (A) G. Torner, J. Booth, (B) M. ‘Evolving Comprehensive VA Women’s Health Care: Patient Characteristics, Needs, and Preferences’	2011	US	1002 women veterans*, enrolled in VHA care	Any veteran services, including statutory (VHA), third sector, community, or private.	Quantitative: Cross sectional survey	Gender-sensitive care for women.
Moin, T. Schneider, J. Vasti, E. Makki, F. Richardson, C. Havens, K. Damschroder, L. ‘Women Veterans’ Experience with a Web-Based Diabetes Prevention Program: A Qualitative Study to Inform Future Practice’	2015	US	15 women veterans with pre-diabetes**, enrolled in VHA care	Statutory (VHA)	Qualitative: Semi-structured interviews	Accessibility
Montgomery, E. ‘Boots and heals: a qualitative study of women veterans’ experiences at veteran health care facilities.’	2021	US	8 female/women veterans**, enrolled in VHA care	Statutory (VHA)	Qualitative: Semi-structured interviews	Discomfort in men-centric environments. Gender-sensitive care for women. Accessibility Service user-provider relationship
Monteith, L. L. Bahraini, N. H. Gerber, H. R. Dorsey Holliman, B. Schneider, (A) L. Holliday, R. and Matarazzo, (B) B. ‘Military sexual trauma survivors’ perceptions of veterans health administration care: A qualitative examination.’	2020	US	50 veterans (32 women, 18 men*) with history of Military Sexual Trauma	Statutory (VHA)	Qualitative: Semi-structured interviews	Help-seeking stigma Service user-provider relationship Continuity/integration of care Discomfort in men-centric environments. Gender-sensitive care for women. Negative military experiences
Monteith, L. L. Holiday, R. Schneider, L. A. Miller N. C. Bahraini, H. N. and Forster, E. J. ‘Institutional Betrayal and Help-Seeking Among Women Survivors of Military Sexual Trauma’	2021	US	242 women veterans** with experience of Military Sexual Trauma	Any veteran services, including statutory (VHA), third sector, community, or private.	Quantitative: Cross-sectional survey	Negative military experiences
Moreau, J. L. Dyer, K. E. Hamilton, A. B. Golden, R. E. Combs, A. S. Carney, D. V. Frayne, S. M. Yano, E. M. Klap, R. and VA ‘Women Veterans’ Perspectives on How to Make Veterans Affairs Healthcare Settings More Welcoming to Women.’	2020	US	1303 women veterans*, enrolled in VHA care.	Statutory (VHA)	Quantitative: Cross sectional survey	Gender-sensitive care for women. Gender discrimination against women from service providers Discomfort in men-centric environments. Peer support
Murray-Swank, N. Dausch, B. M. and Ehrnstrom, C. ‘The mental health status and barriers to seeking care in rural women veterans.’	2018	US	101 women veterans*, enrolled in VHA care, residing in rural areas	Statutory (VHA)	Quantitative: Cross sectional survey	Accessibility Help-seeking stigma Discomfort in men-centric environments. Awareness of services and eligibility
Nash, P.V.J., ‘Guided Exploration of Military Servicewomen’s Healthcare Experiences and Related Outcomes.’	2022	US	321,682 female veterans* from nationally representative survey, 20 African American female veterans* for interview	Statutory (VHA)	Mixed methods: Cross sectional survey & semi-structured interviews	Intersectionality Service user-provider relationship Accessibility
Newins, A. R. Wilson, S. M. Hopkins, T.A. Straits-Troster, K. Kudler, H. and Calhoun, P. S. ‘Barriers to the use of Veterans Affairs health care services among female veterans who served in Iraq and Afghanistan’	2019	US	186 female veterans**, served in Afghanistan and/or Iraq conflicts.	Statutory (VHA)	Quantitative: Cross sectional survey	Help-seeking stigma Accessibility Awareness of services and eligibility
Nicosia, F. M. Gibson, C. J. Purcell, N. Zamora, K. Seal, K. H. ‘Women Veterans’ Experiences with Integrated, Biopsychosocial Pain Care.’	2021	US	14 women veterans* enrolled in VHA pain management sessions.	Statutory (VHA)	Qualitative: Semi-structured interviews	Gender-sensitive care for women. Accessibility
Olmos-Ochoa, T.T., Speicher, S., Ong, L.E., Kim, J., Hamilton, A.B. and Cloitre, M. ‘Supporting equitable engagement and retention of women patients in a trauma-informed virtual mental health intervention: Acceptability and needed adaptations.’	2023	US	26 women veterans* from racial and ethnic minority backgrounds	Statutory (VHA)	Qualitative: Semi-structured interviews	Peer support Help-seeking stigma
Owens, G. P. Herrera, C. J. and Whitesell, A. A. ‘A preliminary investigation of mental health needs and barriers to mental health care for female veterans of Iraq and Afghanistan.’	2009	US	50 female veterans* who served in the Iraq and/or Afghanistan conflicts	Any veteran services, including statutory (VHA), third sector, community, or private.	Quantitative: Cross sectional survey	Help-seeking stigma Discomfort in men-centric environments.
Pebole, M. M. VanVoorhees, E. E. Chaudhry, N. Goldstein, K. M. Thompson, J. Parker, R. Caron, K. M. and Hall, K. S. ‘Patient-centred behavioural services for women veterans with mental health conditions’	2021	US	107 women/female** veterans enrolled in VHA mental healthcare.	Statutory (VHA)	Quantitative: Cross sectional survey	Gender-sensitive care for women. Accessibility
Perry, L.K., ‘Perceptions of Female Veterans Regarding Mental Health Services in the Veterans Administration System.’	2023	US	14 female veterans*, enrolled in VHA care for PTSD and combat exposure	Statutory (VHA)	Qualitative: Semi-structured interviews	Accessibility Gender discrimination against women from service providers Gender-sensitive care for women.
Possemato, K. Wray, L. O. Johnson, E. Webster, B. and Beehler, G .P. ‘Facilitators and Barriers to Seeking Mental Health Care Among Primary Care Veterans With Posttraumatic Stress Disorder’	2018	US	27 veterans (4 female veterans*), enrolled in VHA care for PTSD	Statutory (VHA)	Mixed methods: Focus groups & cross-sectional survey	Gender discrimination against women from service providers
Rohs, C.M., Albright, K.R., Monteith, L.L., Lane, A.D. and Fehling, K.B., ‘Perspectives of VA healthcare from rural women veterans not enrolled in or using VA healthcare.’	2023	US	29 rural women veterans*, not enrolled in or not using VHA care	Statutory (VHA)	Qualitative: Focus groups	Awareness of services and eligibility Gender-sensitive care for women. Accessibility
Rose, D.E., Oishi, S.M., Farmer, M.M., Bean-Mayberry, B., Canelo, I., Washington, D.L. and Yano, E.M., ‘Association Between Availability of Women’s Health Services and Women Veterans’ Care Experiences.’	2022	US	4254 women veterans*, enrolled in VHA care	Statutory (VHA)	Quantitative: Cross sectional survey	Accessibility Service user-provider relationship Gender-sensitive care for women.
Rossi, F. S. Javier, S. J. Kimerling, R. ‘An Examination of the Association Between Patient Experience and Quality of Mental Health Care Among Women Veterans’	2021	US	6287 women veterans*, enrolled in VHA care	Statutory (VHA)	Quantitative: Cross sectional survey	Gender discrimination against women from service providers Gender-sensitive care for women.
Shamaskin-Garroway, A. Knobf, T. M. Haskell, S. G. ‘I Think It’s Pretty Much the Same, as It Should Be”: Perspectives of Inpatient Care Among Women Veterans’	2018	US	25 women veterans, admitted to inpatient care at VHA facilities	Statutory (VHA)	Qualitative: Semi-structured interviews	Gender-sensitive care for women. Service user-provider relationship
Sheahan, K. L. Goldstein, K. M. Than, C. T. Bean-Mayberry, B. Chanfreau, C. C. Gerber, m. r. Rose, D. E. Brunner, J. Canelo, I. A. Darling, J. E. Haskell, S. Hamilton, A. B. Yano, E. M. ‘Women Veterans’ Healthcare Needs, Utilization, and Preferences in Veterans Affairs Primary Care Settings’	2022	US	1391 women veterans with 3 or more visits to VHA facility in the past 12 months	Statutory (VHA)	Quantitative: Cross sectional survey	Gender-sensitive care for women.
Shipherd, J. C. Darling, J. E. Klap, R. S. Rose, D. and Yano, E. M. ‘Experiences in the Veterans Health Administration and Impact on Healthcare Utilization: Comparisons Between LGBT and Non-LGBT Women Veterans’	2018	US	1391 female/women** veterans, enrolled in VHA care	Statutory (VHA)	Quantitative: Cross-sectional survey	Discomfort in men-centric environments.
Shoemaker, M. von Hlatky, S. “Unblurring the Lines of Responsibility: The Puzzle of Veteran Service Provision and its Gendered Implications”	2020	Canada	52 veterans* (22 women and 30 men).	Any veteran services, including statutory, third sector, community, or private.	Qualitative: Focus groups	Negative military experiences Discomfort in men-centric environments. Peer support.
Silverstein, N. M. Moorhead, J. L. ‘Responding to social service and health care needs of aging women veterans’	2001	US	220 women veterans*, over 60 years, community sample.	Statutory (VHA)	Quantitative: Cross sectional survey	Gender-sensitive care for women. Veteran identity Awareness of services and eligibility Accessibility Continuity/integration of services Service user-provider relationship
Silvestrini, M. Nicosia, F. Spar, M. J. Gibson, C. J. and Brown, R. T. (2020) ‘“We Have a Long Way to Go:” A Case Study Examination of Older Women Veterans’ Experiences in VA Primary Care’	2020	US	2 women veterans*, over 60 years, enrolled in VHA care.	Statutory (VHA)	Qualitative: Case study	Gender-sensitive care for women. Gender discrimination against women from service providers
Street, A. E., Shin, M. H., Marchany, K. E., McCaughey, V. K., Bell, M. E., & Hamilton, A.B. ‘Veterans’ perspectives on military sexual trauma-related communication with VHA providers’	2019	US	55 veterans (28 women and 27 men*), enrolled in VHA care, with experience of MST	Statutory (VHA)	Qualitative: Semi-structured interviews	Service user-provider relationship Accessibility Gender-sensitive care for women.
Than, C. T. Washington, D. L. Vogt, D. Chuang, E. Needleman, J. Canelo, I. Meredith, L. S. and Yano, E. M. ‘Discontinuity of Women Veterans’ Care in Patient-Centered Medical Homes: Does Workforce Gender Sensitivity Matter?’	2022	US	9958 women veterans*, enrolled in VHA care.	Statutory (VHA)	Quantitative: Cross sectional survey	Gender-sensitive care for women.
Thomas, K. H. Haring, E. L. McDaniel, J. Fletcher, K. L. and Albright, D. L. ‘Belonging and Support: Women Veterans’ Perceptions of Veteran Service Organizations’	2017	US	829 female/women** veteran members of charitable organisation	Third sector/charitable organisation	Quantitative: Cross sectional survey	Discomfort in men-centric environments.
Trompeter, N., Rafferty, L., Dyball, D. et al. ‘Gender differences in structural and attitudinal barriers to mental healthcare in UK Armed Forces personnel and veterans with self-reported mental health problems.’	2023	UK	1448 veterans (219 women), representative national sample	Any veteran services, including statutory (NHS), third sector, community, or private.	Quantitative: Cross sectional survey	Help-seeking stigma
True, G. Rigg, K. K. Butler, A. ‘Understanding Barriers to Mental Health Care for Recent War Veterans Through Photovoice’	2014	US	40 veterans*** who had served in the Iraq and/or Afghanistan conflicts	Statutory (VHA)	Qualitative: Photovoice and semi-structured interviews.	Gender discrimination against women from service providers
Tsai, J. David, D. H. Edens, E. L. and Crutchfield, A. ‘Considering childcare and parenting needs in Veterans Affairs mental health services’	2013	US	147 veterans (132 males, 15 females)**, enrolled in VHA mental healthcare	Statutory (VHA)	Quantitative: Cross sectional survey	Accessibility
Tsai, J. Mota, N. P. and Pietrzak, R. H. ‘U.S. Female Veterans Who Do and Do Not Rely on VA Health Care: Needs and Barriers to Mental Health Treatment’	2015	US	1202 veterans, including 105 female veterans** using VHA, 373 female veterans using other healthcare, and 724 men using VHA care	Any veteran services, including statutory (VHA), third sector, community, or private.	Quantitative: Cross sectional survey	Accessibility Help-seeking stigma
Tuepker, A. Newell, S. Sorrentino, A. Cusack, M. True, G. & Dichter, M. E. ‘High-Risk Encounters: Primary Care Experiences of Women Living with Intimate Partner Violence, and Implications for the Patient Centred Medical Home’	2022	US	50 women veterans*, experienced IPV in past 12 months, enrolled in VHA care	Statutory (VHA)	Qualitative: Semi-structured interviews	Service user-provider relationship
Turchik, J. a. Bucossi, M. M. Kimerling, R. ‘Perceived Barriers to Care and Gender Preferences Among Veteran Women Who Experienced Military Sexual Trauma: A Qualitative Analysis’	2014	US	9 female veterans* with experience of MST, enrolled in VHA care	Statutory (VHA)	Qualitative: Semi-structured interviews	Help-seeking stigma Service user-provider relationship Discomfort in men-centric environments. Awareness of services and eligibility Gender-sensitive care for women.
Vogt, D. Bergeron, A. Salgado, D. Daley, J. Ouimette, P. and Wolfe, J. ‘Barriers to Veterans Health Administration care in a nationally representative sample of women veterans’,	2006	US	942 women veterans*, current and former users of VHA care	Statutory (VHA)	Quantitative: Cross Sectional survey	Gender-sensitive care for women. Accessibility Continuity/integration of care
Wagner C, Dichter ME, Mattocks K. ‘Women Veterans’ Pathways to and Perspectives on Veterans Affairs Health Care.’	2015	US	249 women veterans*, past users of VA care for survey. Sub-set of 25 women veterans* for interview.	Statutory (VHA)	Mixed methods: Cross sectional survey and semi-structured interviews	Accessibility Awareness of services and eligibility Negative military experiences Gender-sensitive care for women. Discomfort in men-centric environments. Peer support
Ward, C. J. Cope, M. R. and Jackson, J. ‘Healthcare Access among Older Rural Women Veterans in Utah’	2020	US	22 older women/female veterans**, residing in rural areas	Any veteran services, including statutory (VHA), third sector, community, or private.	Qualitative: Focus groups	Awareness of services and eligibility Accessibility Discomfort in men-centric environments. Gender discrimination against women from service providers
Washington, D. L. Yano, E. M. Simon, B. and Sun, S. ‘To use or not to use: What influences why women veterans choose VA health care’	2006	US	2174 women veterans*, eligible for VHA care	Statutory (VHA)	Quantitative: Cross-sectional survey	Gender-sensitive care for women. Accessibility Awareness of services and eligibility
Washington, D. L. Kleimann, S. Michelini, A. N. Kleimann, K. M. Canning, C. ‘Women Veterans’ Perceptions and Decision-Making about Veterans Affairs Health Care’	2007	US	51 women veterans*, eligible for VHA care	Statutory (VHA)	Qualitative: Focus groups	Awareness of services and eligibility Gender-sensitive care for women.
Washington, D. L. Bean-Mayberry, B. Riopelle, D. Yano, E.M. ‘Access to care for women veterans: Delayed healthcare and unmet need’	2011	US	3611 women veterans*, national representative survey	Statutory (VHA)	Quantitative: Cross sectional survey	Gender-sensitive care for women. Awareness of services and eligibility
Washington, D. L. Farmer, M. M. Mor, S. S. Canning, M. Yano, E. M. ‘Assessment of the Healthcare Needs and Barriers to VA Use Experienced by Women Veterans’	2015	US	3611 women veterans*, national sample	Any veteran services, including statutory (VHA), third sector, community, or private.	Quantitative: Cross sectional survey	Accessibility Awareness of services and eligibility
Williams, L. ‘Women Veterans’ Perceptions of Mental Health Outpatient Services’	2015	US	12 women veterans*, enrolled in VHA and non-VHA mental healthcare	Any veteran services, including statutory (VHA), third sector, community, or private.	Qualitative: Semi-structured interviews	Discomfort in men-centric environments.
Willett, C., ‘Lived Experience of Women Veterans Navigating the Veteran Healthcare System.’	2023	US	12 women veterans*, enrolled in VHA care	Statutory (VHA)	Qualitative: Semi-structured interviews	Gender-sensitive care for women. Gender discrimination against women from service providers Accessibility
Wolgemuth, T. E. Cuddeback, M. Callegari, L. S. Rodriguez, K. L. Zhao, X. and Borrero, S. ‘Perceived barriers and facilitators to contraceptive use among women veterans accessing the Veterans Affairs Healthcare System’	2020	US	189 women veterans*, enrolled in VHA care, at risk of pregnancy.	Statutory (VHA)	Qualitative: Telephone interviews	Awareness of services and eligibility Service user-provider relationship Discomfort in men-centric environments. Gender-sensitive care for women.
Wood, Fossey, Price, Powell, Chalkley, Davidson & Godier-McBard. ‘I don’t feel like that’s for me’. Overcoming barriers to mental healthcare for women veterans.	2023	UK	48 women veterans, with self-reported mental health conditions	Any veteran services, including statutory (NHS), third sector, community, or private.	Qualitative: Semi-structured interviews	Help-seeking stigma Gender discrimination against women from service providers Gender-sensitive care for women. Negative military experiences Awareness of services and eligibility Veteran identity Service user-provider relationship Discomfort in men-centric environments.
Yeager, J ‘The Experiences of Female Student Service Members and Veterans as Depicted Through Photovoice’	2022	US	9 female veterans*, college students	University veteran-specific support services	Qualitative: Photovoice, focus groups, & semi-structured interviews	Peer support
Zickmund, S. L. Burkitt, K. H. Gao, S. S. Stone, R. A. Jones, A. L. Hausmann, L. R. M. Switzer, G. E. Borrero, S. Rodriguez, K. L. Fine, M. J. ‘Racial, Ethnic, and Gender Equity in Veteran Satisfaction with Health Care in the Veterans Affairs Health Care System’	2018	US	1222 veterans (616 female veterans)**, enrolled in VHA care	Statutory (VHA)	Quantitative: Cross sectional survey	Peer support

*Gender or sex terms used without definition or explanation of eligibility for participation (i.e. no explanation of whether participants were included based on biological sex or gender identity)

**Gender or sex terms used interchangeably in relation to sample, without definition or explanation of eligibility for participation

*** Results stratified by sex or gender, but sample size of each group not provided

## Results

**Fig. 1:**
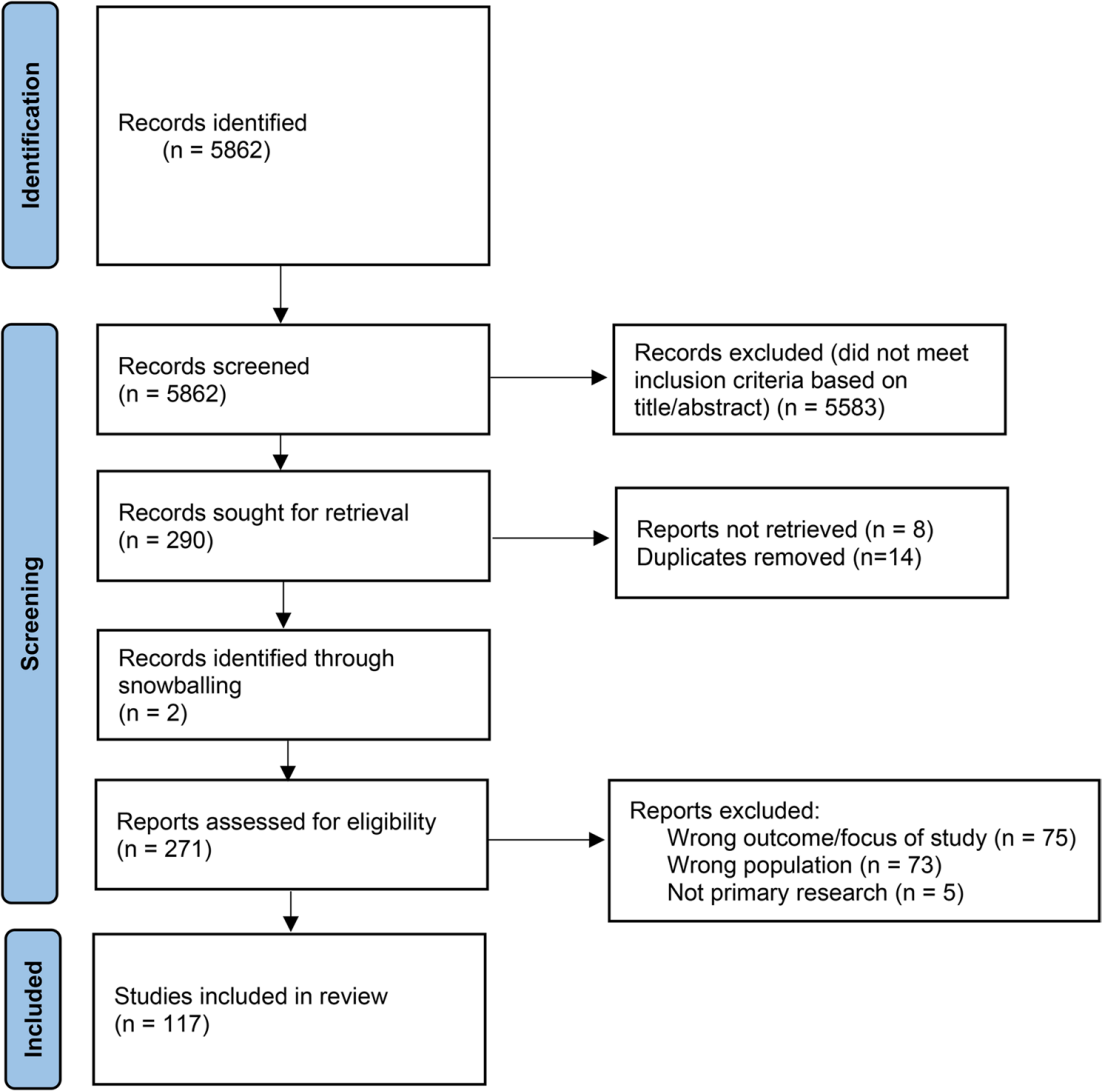
PRISMA diagram

The search yielded a total of 5,862 records. Following a review of the titles and abstracts, 5583 papers were removed as being assessed as not relevant to the study research questions or not meeting the inclusion criteria (see Table 1). Following removal of duplicates and inaccessible records (this included conference abstracts and texts the authors could not access via either inter-library loans or contacting authors), 268 full-text papers remained. Following a full text review 88 papers remained. An additional 29 records that met the inclusion criteria were identified through backward and forward reference searching. Two additional papers were identified during the peer review process. A total of 117 articles were included in the review. This process is outlined in Fig. 1 (PRISMA diagram).

The research reported in the reviewed papers spans several different geographical, political, social, and health provision perspectives. These dimensions need to be taken into consideration in any conclusion drawn in the various studies. Whilst many of the findings are universal, the prevailing culture of support and provision needs to be considered.

### Study characteristics

Table 2 outlines the characteristics and themes identified in each paper. Eight papers originated from the UK, 108 from the US, and one from Canada. Sixty-three papers used qualitative methods, 46 papers used quantitative methods, and 8 papers used mixed methods. Two UK-based papers focused on experiences of third sector (charitable) veteran support services only, and the remaining 6 papers focused on any veteran services, including third-sector support services, private healthcare, and healthcare provided by the National Health Service (NHS). Of the papers originating from the US, 2 studies focused on non-Veterans Affairs (VA) veteran-specific services only (i.e. community support & third sector/charitable veteran organisations that serve only veterans), and 20 included both VA and any other support services (i.e. including community services, third sector, and private healthcare open to both veterans and civilians). The remaining 87 US studies centered solely on the Veterans Health Administration (VHA) services. The sole Canadian paper focuses on experiences of any veteran services, including statutory, third sector, community, or private. Participant characteristics varied across studies including help-seeking only, mixed help-seeking and non-help-seeking, “rural” veterans only, Native American veterans only, and veterans of specific military operations (i.e. veterans who served in the Iraq and Afghanistan wars).

Most of the papers (77 out of 117) used gender terminology to describe study participants (i.e. “women veterans”), with just 16 using terminology associated with biological sex only (i.e. “female veterans”). However, 24 papers used sex and gender terms interchangeably to describe their participants (i.e., “female/women veterans”), without providing a definition or explanation of their use of these terms or eligibility for participation. Notably, just 8 papers provided an explicit explanation of their use of gender/sex terminology and/or eligibility for participation based on sex/gender identity.

We identified 11 themes from the existing literature that represent the experiences of accessing veteran-specific care for women veterans, outlined in Table 3. The themes predominantly represent barriers and enablers to accessing veteran-specific care and preferences of what support should look like for women within veteran services. Many of the barriers discussed were also mentioned conversely in the context of enablers i.e. a lack of, and therefore a need for, gender-sensitive services for women, and as such the themes are not split into barriers and facilitator sections.

**Table 3:** Summary of themes

Theme	No of papers (out of 117)	Outline of theme
Gender sensitive care for women	67	Women veterans want services and service providers that understand their gendered experiences during military service and unique support needs after service. The availability of women’s health clinics, women-only settings, women service providers, and women-only support/treatment groups is reported. Evidence supports the effectiveness of providing gender-sensitive care in meeting women veterans care needs.
Accessibility	54	Women veterans reporting practical barriers to accessing care, including mismanaged appointments, long wait times, poor signposting, distance from facilities, high treatment costs, difficulties organising childcare, and the complex nature of healthcare systems. Telehealth is suggested to overcome some of these barriers.
Discomfort in men-centric environments.	38	Women veterans report discomfort and feeling unwelcome when accessing men-centric veteran services, which are perceived to have been designed around the needs of men, and predominantly accessed by men. UK studies highlight the dominance of men in imagery and language in service branding, which is off-putting to women. Harassment by veteran men on VA grounds is reported by women, impacting their willingness to return to access further care.
Service user-provider relationship	34	Women veterans want service providers who are empathetic, compassionate and understanding, with many reporting experiences of feeling dismissed, unheard and judged by service providers. Positive service user-provider relationships are associated with a better experience of support for women veterans. Women veterans want involvement in decisions about their care.
Awareness of services & eligibility	28	Women veterans report confusion about the eligibility criteria for accessing veteran-specific care and a lack of knowledge about the services that are available to them, which acts as a barrier to accessing care.
Gender discrimination from service providers	24	Women veterans report feeling discriminated against or treated differently by veteran services because of their gender, including their veteran status being questioned. This includes feeling their experiences or health concerns are misunderstood, dismissed, minimised, or disbelieved.
Help-seeking stigma	23	Women veterans report stigma associated with seeking help that is cultivated during military service, and embarrassment and shame when asking for help. Most gender-comparisons suggest no gender-based differences in help-seeking stigma amongst veterans. However, women veterans report an additional layer of stigma, associated with discriminatory attitudes regarding the perceived weakness of women in military service.
Peer support	22	Women veterans wanted access to peer support and highlighted the importance of interacting with other women veterans to help them navigate and engage with support services, and to reduce feelings of isolation.
Negative military experiences	17	Women veterans report the impact of adverse experiences whilst serving in the military on their willingness to seek support from veteran organisations. This is particularly pertinent for those with experience of military sexual trauma (MST), for whom these services were perceived as reminiscent of the military environment. Women veterans also report that negative experiences of seeking support during service were related to reluctance to access veteran support services.
Continuity/integration of services	14	Poor continuity of care and a lack of integration between and within services was reported by women veterans as a barrier to engaging with support. This included poor linkage from military to veteran services, poor coordination between veteran and non-veteran support services, and having to repeat traumatic stories due to service provider not reviewing previous medical notes.
Veteran identity	10	Women veterans report a lack of identification with the term ‘veteran’ which impacts the likelihood of seeking out and accessing veteran-specific services. Additionally, women veterans report not always being asked if they have served in the military by support services, leading to them missing out on veteran-specific care.

### Theme 1: gender-sensitive care for women

The importance of gender sensitivity within veteran support services is a key enabler to access cited by women veterans in over half (67/117) of the papers identified in this review [6, 7, 27–92]. Across these papers, women veterans want support services that have a good understanding of their gendered experiences and needs as ex-servicewomen and the expertise to meet these needs. This includes a broader understanding of women’s health. Indeed, lower gender sensitivity from service providers is associated with discontinuation of engagement with VA service in women veterans [82].

Several papers highlight that women veterans often face unique challenges during and after military service, including being more at risk of exposure to military sexual trauma (MST), and as such have distinct healthcare needs compared to veteran men [58, 83, 92]. For example, those who experience MST are more likely to report a preference for a woman clinician [83]. Additionally, women have different healthcare needs to men regardless of veteran status, including being more at risk of certain health conditions and factors that impact health [39, 59]. Women veterans in the US and UK report that their specific support needs have not yet been fully embraced by veteran healthcare services [6, 7, 58].

Studies across the US and UK highlight the need for veteran service providers to receive specific training around the unique experiences and needs of women, and the need for more gender-specific services for women veterans [6, 7, 32, 74]. In one survey, US women veterans suggest that additional training on gender bias, prejudice against women veterans, and gender sensitivity training would support better care [63].

Many US studies identify the availability of women’s health clinics within the VHA as a factor that could enable access to and satisfaction with care [35, 51, 58, 62, 63, 76, 77, 84, 87, 88]. Furthermore, whilst not universal, a preference for women-only settings, women service providers, and women-specific support/treatment groups is evident across US and UK studies, with women feeling more comfortable being supported by and interacting with other women [6, 7, 29, 30, 37, 49, 50, 54, 56, 72, 73, 78, 90, 91]. This was particularly important for those who have experienced MST, for whom the men-centric nature of veteran services caused discomfort [7, 36, 55, 58, 67]. Indeed, one survey found that provider gender mismatch (i.e. service user and provider are different genders) was associated with a greater endorsement of barriers to care, less comfort in the support environment, and provider competency for women veterans [67]. Women veterans also report a preference for women-only waiting areas and inpatient units [56, 68, 77].

The reviewed evidence supports the effectiveness of gender-sensitive/specific care in meeting the needs of women veterans [65]. Women veterans in Fitzke et al.’s [44] study report increased satisfaction with VA healthcare because of improvements to women-specific services, including the availability of women service providers, women-only therapy groups, and gender-specific healthcare services. Kimerling et al. [54] found that gender-related experiences, including the availability of women service providers and women-only treatment settings and groups, were each associated with 2-fold increased odds of perceived access to care. Access to gender-specific services is associated with greater trust in the VA [33] and reduced odds of perceiving gender-based discrimination within services [62]. However, some studies report that women veterans have concerns about the ability of veteran services to provide support for female-specific health conditions, including reproductive health, when they have been designed around the needs of male veterans [49].

## Theme 2: accessibility

The accessibility of veteran services is a key barrier to care for women veterans cited in 54 papers [6, 28, 30–32, 35, 37, 39–43, 46–49, 51, 52, 54, 59, 70, 72–76, 79, 81, 84, 85, 88, 89, 92–113]. Inconvenient appointment times, mishandled appointments, and scheduling conflicts are frequently cited [7, 30, 41, 73, 74, 89], leading to difficulties accessing services promptly. Poor signposting and handover to VA care following service acts as a barrier to women veterans utilizing such services [40, 112]. Long wait times for appointments impeding access to services are also reported across studies [7, 39, 41, 46, 49, 58, 84, 102]. Women veterans report difficulties navigating complex healthcare system [74] across countries [7, 31, 32, 113].

The location of, and travel distance to, veteran services is identified by women veterans as a salient barrier to accessing care [6, 7, 38, 46, 47, 73, 97, 107, 111]. This is also highlighted as a key barrier for US women veterans living in rural areas [30] and Native American women veterans living on reserves [28]. Friedman et al. [95] report that longer drive times are associated with higher odds of attrition from VA care for women veterans. The VA shuttle system, which provides transport between facilities, is also identified as an enabling factor for accessing services by women veterans [39]. In the UK, women veterans report a postcode lottery in the availability of veteran services [7, 46].

High treatment costs are identified as a barrier to accessing care for women veterans in several US studies [28, 92, 101, 105, 107]. Tsai et al. [109] report that 25% of women veterans in their study endorse high treatment costs as a barrier to accessing VA mental health care. Conversely, affordability of VA care is also reported by women veterans as an enabling factor to accessing services [35, 88].

Childcare responsibilities are identified as a factor negatively impacting access to veteran support services in the US and the UK, with women veterans reporting that family responsibilities affect their ability to schedule and attend appointments [6, 30, 43, 73, 105]. Within three studies, women veterans report that on-site childcare would be an enabling factor for accessing care [37, 42, 108]. It should be noted that one study reports that both veteran women and men highlight childcare as a barrier to accessing support services [108].

The use of telehealth technology is reported in several studies as a way of overcoming accessibility barriers [30, 39, 104]. Women veterans living in rural areas report that telehealth can enable their access to care by reducing travel times and allowing for more consistency in service providers [30]. However, online healthcare is seen as difficult to access by older women veterans [110].

### Theme 3: discomfort in men-centric environments

The impact of accessing men-centric veteran care settings is identified as a barrier to accessing services by women veterans in 38 papers [6, 7, 14, 29–32, 36, 37, 39, 40, 42, 44, 45, 51, 56, 58, 69–71, 83, 85, 90, 91, 96, 105, 110, 113–123]. There was a perception that these services had been set up for and designed around men, were predominantly accessed by men, and were ill-equipped to meet the needs of women [6, 7, 30–32, 40, 49, 80, 123]. Women veterans report feeling ‘stared at’ by men while waiting for appointments and that this made them feel a sense of not belonging at VA facilities [30]. Furthermore, women veterans report that this male-centric environment is reminiscent of the military [89]. Four of these studies investigate the experiences of women with a history of MST, with women veterans reporting that they are not comfortable in mixed-gender (predominantly men) veteran support groups and feel unsafe in mixed-gender inpatient accommodations [7, 30–32, 69, 118]. Two studies identified provider gender as a barrier to care for women veterans: In a survey study, McBain et al. [67] found that provider-patient gender mismatch is associated with less comfort with service providers. In a qualitative study, McDonald et al. [91] found that women veterans described feeling unsafe or on guard when disclosing trauma to clinicians who were men.

Not feeling ‘welcome’ as women within men-centric veteran-specific services is reported as a salient barrier to accessing services [14, 30, 89, 90, 116, 118, 121, 122]. US-based surveys report that the VHA environment is ‘unwelcoming’ to women veterans [117, 121, 122]. In Thomas et al.‘s [14] survey of women veterans accessing veteran service organisations and charities, 30% of respondents identified not feeling welcome in veteran groups as one reason that they weren’t currently members of such organizations. Research in the UK echoes this, with 20% of women veterans in one study reporting that they don’t feel comfortable accessing veteran organisations for support [114]. Qualitative research suggests that this discomfort in the VHA environment is related to the men-centric nature of these services [7, 89, 118]. Conversely, comfort in the treatment environment is found to be an enabler of attending, engaging in, and completing treatment [7, 117, 124].

Women veterans across US studies report experiencing harassment from veteran men on VA grounds, impacting their willingness to attend these settings again [44, 50, 58, 70, 71, 116, 120, 122, 123]. Klap et al. [120] found that 1 in 4 women veterans report experiencing inappropriate or unwanted comments or behavior on VA grounds, such as sexual or derogatory comments. Furthermore, women veterans who experience harassment are significantly less likely to feel welcome at the VA and more likely to delay or miss out on care. LGBTQ+ women veterans report that the harassment they experience from men on VA grounds is distressing, and has influenced their ‘healthcare accessing behavior’ [122].

### Theme 4: service user-provider relationship

The service user-provider relationship is an important factor influencing women veterans’ experiences within veteran services, discussed in 34 studies [7, 33, 36, 38–40, 42, 43, 45, 49, 51, 52, 56, 58, 61, 69, 70, 76, 77, 79, 81, 83, 89–100, 106, 115, 124–127]. A lack of empathy and understanding from veteran healthcare providers is reported by women veterans in several studies [46, 89]. This can impact on the quality of care and patient satisfaction, and subsequent willingness to seek care [89]. Women veterans report feeling judged, shamed, and dismissed by veteran healthcare providers [7, 44, 99, 115]. However, it is worth noting that one study [125] of over 55,000 veterans reported positive perceptions of VA psychiatric care (over 90% of women veterans agreed that they felt listened to, involved in decisions about their care, and trusted their provider), which did not differ between genders.

Positive service user-provider relationships are cited as key to providing sensitive, respectful, and patient-centered care to women veterans and promoting positive outcomes [7, 49, 81, 99, 106, 124, 126]. The development of trust within service user-provider relationships, including being reliable, showing understanding, and respecting autonomy is identified by women veterans as a key enabler to trauma disclosure and quality care [7, 99, 124]. Indeed, one US study reports that trust in the VA is associated with greater use of services by women veterans [94]. Clear and effective communication from service providers is highlighted as vital [74, 81], but often lacking [89].

Choice and agency in treatment decisions is also identified as key to supporting women veterans to engage in treatment [7, 38, 69, 123]. For UK women veterans, this includes choice of clinician gender and military background, and the option to access gender-specific care settings [7]. US women veterans express a preference for the choice of holistic options and different treatment programs [36, 69, 123]. A proactive approach from service providers is desired by UK women veterans [7, 31, 32], both in initial and continued engagement in services.

### Theme 5: awareness of services and eligibility

Confusion around eligibility for veteran-specific care, and a lack of knowledge about the types of services available is reported as a key barrier to accessing care for women veterans in 28 papers [6, 7, 28, 37, 40, 48, 56, 59, 66, 75, 79, 83, 85–88, 90–93, 101, 103, 105, 110–112, 128, 129].

Women veterans report being unsure of whether they are eligible to access veteran services [103]. In the US, Washington et al. [86] report that almost half of the women veterans in their survey did not know they were entitled to access VA care (49.4%). In the UK, women veterans report being unsure of the eligibility based on having ‘service-attributable’ conditions required to access NHS-based veteran services, and a perception that veteran-specific services are for those with combat-related injury or illness [7].

A lack of awareness and knowledge gaps around veteran-specific care is a salient barrier across literature. Conversely, Arnold [128] reports that awareness of VA services is associated with increased utilization. Across many qualitative studies, women veterans specifically mention a lack of awareness of gender-specific/women-only veteran services [37, 66, 87, 90, 110], and women veterans who did not use the VA’s services often assumed the VA did not provide women’s healthcare [87].

### Theme 6: gender discrimination from service providers

Gender stereotyping and gender-based discrimination is reported by women accessing veteran support services in 24 identified papers [7, 15, 27, 36, 39, 43, 46, 48, 51, 62, 63, 66, 71, 74, 80, 89, 93, 96, 110, 127, 130–132]. Women veterans report feeling discriminated against, treated differently, or receiving lower-quality care because of their gender [40, 44, 46, 74, 93, 127, 130–132]. Furthermore, women veterans perceived that they had a lower status than men in the VA and that this affected their medical care [110]. In the US, women veterans report that when accessing the VA, assumptions were made due to their gender, including their veteran status being questioned by patients and staff, and people assuming they are wives or family members of veterans rather than ex-service personnel accessing care for themselves [40, 93, 132]. Indeed, Macdonald et al., [62] found that one-third of women veterans perceived there to be gender-based discrimination in the VA.

Women veterans report that their health issues are often misunderstood, minimized, dismissed, or disbelieved because of their gender [7, 31, 32, 39, 46, 58, 63, 96]. Women veterans report feeling as though veteran care providers are designed to cater to men, have little understanding of how women’s health needs may differ, and that as a result women’s needs were often not met within these services [6, 7, 31, 32, 39, 40, 49, 61, 80, 110]. Additionally, women report a lack of recognition that women may have experienced the same trauma as men in service [15]. In some cases, women felt service providers did not believe or take their symptoms seriously because of their gender and reported being told that their symptoms or conditions were due to female-specific hormonal fluctuations or their weight [39, 63]. Women veterans reported that this perception of gender discrimination within services had shaped how they view the VA [60], particularly for women veterans with a history of MST [62].

### Theme 7: help-seeking stigma

The impact of help-seeking stigma cultivated during military service is highlighted in 23 studies as a barrier to accessing support for women veterans [6, 7, 15, 28, 31, 32, 43, 46, 49, 56, 69, 83, 99, 105, 107, 109, 121, 129, 130, 133–135). Women veterans suggested that the military culture promotes stoicism, ‘strength’, and self-management of stress, and associates both illness and asking for help with weakness. This contributes to a reluctance to seek support both during and after military service [6, 7, 28, 31, 37, 46, 56, 70, 83, 105, 107, 109, 129, 130, 133]. Women veterans report embarrassment and shame at asking for help [6, 7], fear of repercussions on their career [6], and feeling as though they will be judged by others (including service providers and the military/veteran community) [99]. In one study, almost half of the women veterans with a history of depression or post-traumatic stress disorder had negative treatment biases, including beliefs that one should “work out [one’s] own problems” and that they would be “seen as weak by others” for seeking support [107]. These types of negative beliefs about treatment-seeking are associated with a lower likelihood of seeking care for women veterans [129].

Several studies provide a gender-based analysis of stigma in military veterans. One US study [129] reports that veteran men are more likely to hold negative beliefs about mental illness and mental health treatment than veteran women. For both men and women in this study, negative beliefs about treatment-seeking were associated with decreased likelihood of accessing VA care in this study. However, another US study [109] reports no gender-based difference in stigma-related beliefs as a barrier to accessing care for veterans. Similarly, two UK-based studies report no differences in help-seeking stigma by gender in veterans [15, 134].

However, in addition to the general stigma associated with asking for support, the literature suggests a compounding impact of gender on the stigma that women veterans experience. Many of the UK studies reviewed report that negative and derogatory attitudes towards women during military service, including a perception of ‘female weakness’, compound the stigma felt by women in asking for support [6, 7, 15, 31, 32]. Women in these studies report feeling as though they must prove themselves in service considering negative attitudes towards them, including having to work twice as hard to be judged equally to men. As such, women veterans fear admitting weakness by asking for support.

### Theme 8: peer support

Peer support is identified as a key factor in encouraging access and engagement with veteran support services accessing support services in 22 studies [6, 7, 28, 30–32, 38, 42, 50, 51, 56, 57, 59, 60, 71, 85, 91, 96, 113, 123, 130, 135–137]. Women veterans report the importance of fellow women veterans in helping them navigate veteran-specific services [56, 96] and supporting them to engage with these services [130]. All women veterans in Ingelse and Messcar’s [130] study report that they wouldn’t have sought services without the help of supportive peers. Women veterans also report the importance of friendships with other women veterans in exchanging information about veteran-related support [30]. As such, women veterans would like to see more peer support networks established [6, 57]. Furthermore, access to peer support appears to be associated with decreased feelings of isolation for women veterans [136]. Indeed, in the UK, women veterans reported peer support to be the main contributing factor relating to a sense of belonging within support services [6].

### Theme 9: negative military experiences

The impact of adverse experiences while serving in the military is cited as a barrier to accessing veteran-specific services within 17 papers [7, 15, 30–32, 36, 40, 51, 53, 59, 69, 85, 91, 98, 113, 119, 138]. This is broadly related to the triggering nature of veteran organisations, which women veterans find reminiscent of the military environment and culture within which these negative experiences occurred. For example, in the US context, women veterans report that visiting VA facilities was triggering due to memories of negative experiences while serving, and the proximity of the VA to the military institution [40]. This was particularly pertinent for women veterans with experience of MST, who felt ‘trapped’ by accessing care through an organization with they saw as closely paralleling the military [51]. This is supported by survey research that found that women veterans with histories of MST had poorer perceptions of VHA facilities and staff compared to women veterans who did not [53]. These women veterans related this feeling to a perpetuation of the betrayal they felt from the military [51]. This may be related to feelings of institutional betrayal (IB), defined as when institutions fail to act in ways that prevent harm or enable environments that cause or exacerbate harm, which are also identified as a barrier to seeking veteran specific care [138]. Monteith et al. [138] report that feelings of IB are associated with lower willingness to use VA care and greater willingness to seek non-VA care.

Furthermore, negative perceptions of veteran-specific services are related to negative experiences of support whilst serving within some studies. In the US context, women veterans report a distrust of military-associated doctors that has its origins in bad interactions with healthcare professionals during military service [85]. These experiences act as a barrier to accessing VA services, which are perceived as being military-associated. Similarly, women veterans in the UK report that poor experiences with military-associated support professionals, including feeling dismissed and judged when disclosing traumatic experiences, can negatively impact on willingness to seek support for mental health post-service [7].

### Theme 10: continuity/integration of care

Poor continuity of care and a lack of integration between and within support services is reported as a barrier to accessing and engaging in support in 14 papers [34, 39, 40, 43, 45, 48, 52, 64, 69, 79, 85, 93, 100, 127]. Women veterans report poor coordination and linkage to veteran services after leaving military service, disrupting ongoing care, and obstructing access to new care pathways [6, 40, 43]. Additionally, women veterans report poor coordination between VA and non-VA healthcare facilities and the frustration associated with having to start over with new service providers [39, 48]. For those accessing care through both VA and non-VA facilities, care fragmentation is associated with lower ratings of care quality, whereas those accessing VA-only services report a higher quality of care [34].

Several studies highlight the lack of maternity care in the VA system and the complexity of coordinating care between the VA and outside providers as a result [64, 100]. Indeed, within maternity care, women veterans report that care coordinators are key enablers in helping them navigate VA care and connecting them with outside providers [52]. However, poor care integration with the VA and non-VA providers of maternity care is associated with higher rates of mental healthcare use following pregnancy for women veterans [64]. Women veterans in this study felt that non-VA providers knew little about their medical histories or treatment records from the VA, impacting their care.

Furthermore, women veterans suggest that when engaging with new care providers or clinicians, medical notes are often not reviewed before appointments which impedes care, and means they have to repeat their stories and symptoms multiple times [7, 39, 45, 52]. Several studies suggest that care providers should keep up to date on patient notes before appointments to enable a better care experience [7, 64].

### Theme 11: veteran identity

A lack of identification with the term ‘veteran’ was identified by 10 papers as a barrier to accessing veteran-specific services for women veterans [6, 7, 31, 32, 46, 56, 79, 91, 130, 139]. Women veterans reported not identifying with the term ‘veteran’ for many reasons, including not having served in combat roles [31, 32], not fitting the stereotypical ‘hero’ veteran narrative [6, 7], not feeling as though they deserved the title due to type of role or length of service [6, 56] and other aspects of their identity being more prominent [6, 7]. Additionally, women who have experienced negative gender-based experiences during military service report wanting to distance themselves from the military as an organization, and their veteran status by proxy [7]. Where women veterans are not identifying with this term, they are not likely to seek out services for veterans. Indeed, Di Leone et al. [139] found that the centrality of US women’s veteran identity was positively related to their choice to use VA healthcare, and with their perceived ‘fit’ within the VA and entitlement to use VA care. Furthermore, Ingelse and Messecar [130] found that despite serving in combat, US women veterans did not feel like ‘real’ combat veterans. This perception can then impact on access to services that may use combat-related language in their branding, leading women veterans to question whether these services can meet their needs [6, 7].

Relatedly, women veterans in UK-based studies report that the dominance of men in imagery and branding used for veteran services acts as a barrier to engagement [6, 7]. A similar picture is observed in the US, with Dyer et al. [116] reporting that women veterans feel there is a lack of imagery, statues, and symbols present at VA facilities that represent and include women, which makes them feel that they don’t ‘have a place’ at the VA. In UK literature, this extended to the masculine and military-loaded language used in service branding, for example, the use of the words ‘Op’, and ‘Combat’ in service names, which was off-putting for women veterans, for whom this did not align with their perception of their time in service or identity as a veteran [6, 7].

Additionally, four studies report that women veterans were rarely asked by healthcare providers if they had served in the military, which meant they would not be referred to veteran-specific care [6, 7, 46, 91]. This appears to be a problem specific to the UK context in the available literature, in which veteran healthcare is provided within the broader civilian healthcare system, and service in the armed forces is not recorded as standard.

### Specific women veteran cohorts

The identified papers also highlight the unique experience of women veterans in specific intersectional cohorts, for example, women veterans who live rurally, and those from Native American populations or ethnic minority backgrounds.

Five of the identified papers focus on US women veterans living in rural areas [30, 75, 105, 110, 130]. These studies highlight distance from VA facilities in rural areas [75, 105, 110], and a lack of local services, including dental and mental-healthcare, and a lack of gender-specific services [30, 75, 110, 130] as significant barriers to care. A lack of community-based support is also highlighted, as well as a lack of peer support from other women veterans, due to living in isolated locations [30]. Women veterans suggested an increase in access to Telehealth services to overcome some of these barriers [75].

Two papers focus on the experiences of Native American women veterans [28, 92]. Women veterans in these two studies also highlight barriers to accessing VA care associated with distance to VA facilities, including the cost and availability of transport. Additionally, both studies highlight the role of the Tribal VA representative in supporting them to understand the eligibility criteria and apply for VA support, and barriers associated with the availability of this representative. Many women in these studies chose to utilise Indian Health Services instead of VA care, as a result of these barriers.

Two papers focus on the experiences of US women veterans from non-white ethnic backgrounds [135, 137]. The first of these studies [137] investigated differences in satisfaction with VA services by ethnicity and reports no significant differences. However, this study did find that Black women veterans were less likely to be satisfied with VA services than Black veteran men. The second of these studies provides recommendations for how the VA mental health services could better support women veterans from racial and ethnic minority groups, including improving peer support and developing a community of peers, and diversifying mental health providers [135].

## Discussion

This scoping review sought to understand what is known internationally about the experiences of women veterans accessing veteran-specific support services. Previous reviews have examined women veterans’ support needs and access to care in specific domains, including physical health, mental health, and PTSD [16–20]. This review advances that work by centering women veterans’ experiences of veteran‑specific support in general and by integrating barriers, facilitators, and the conceptualization of sex and gender within a single international synthesis.

Across 117 papers, (predominantly from the US, with smaller bodies of work from the UK and Canada), we identified 11 themes that largely transcended differences in health and social care support systems for veterans across countries. These themes highlighted both gender neutral and gendered influences on access and engagement with services.

Women veterans experience several barriers to accessing support in common with veteran men and the wider public, including stigma in asking for support, accessibility issues, and poor continuity of care or handover of care between services (i.e. either from the military to civilian support system, or between support providers). However, the reviewed literature reveals a series of gendered barriers and enablers to accessing veteran-specific support that are unique to women veterans, and ways in which services could be better tailored to meet their support needs. In focusing on veteran-specific provision and women veterans’ lived experiences, this review extends previous reviews that have concentrated on discrete health outcomes, healthcare utilization, and specific healthcare contexts. Crucially, this review examines how women veterans experience veteran‑specific services as a distinct system of provision, highlighting how gender shapes engagement across the full pathway of access, rather than within isolated domains.

### Gendered barriers to accessing veteran care for women veterans

Our findings suggest that gendered barriers to veteran-specific care are underpinned by a persistence of masculine-normed institutional cultures, and women’s military experiences shape their post-service help-seeking. Women veterans across the literature report the perception that veteran support services have been designed around the default veteran man [6, 7, 30–32, 40, 49, 80, 113, 123]. Feelings of discomfort, invisibility, and discrimination in environments dominated by men [40, 44, 46, 54, 93, 110, 127, 130–132], including having their veteran status questioned [40, 93, 132] or health concerns minimized [7, 31, 32, 39, 46, 58, 63, 96] were reported by women veterans. Whilst this mirrors wider evidence that civilian women’s healthcare needs are frequently underestimated or dismissed by healthcare providers, this appears to be exacerbated in the context of men-centric veteran services [5].

The evidence also shows how gendered stigma and adverse in-service experiences can impact help-seeking. Whilst help-seeking stigma is well-documented in veteran populations [140], studies suggest an additional layer for women, linked to derogatory perceptions of feminine weakness and a pressure to prove themselves in service [6, 7, 15, 31, 32]. For some women with experiences such as MST and institutional betrayal [51, 138], the proximity of veteran services to the military institution that harmed them can contribute to a preference for non-veteran services. This may begin to explain findings that indicate women veterans are more likely to access mainstream or non-veteran-specific healthcare services compared to men [15].

Finally, lack of identification with the term “veteran” appears to be a distinctive barrier for women. Women report that their roles, length of service and negative gendered experiences undermined their sense of being entitled to this label [6, 7, 31, 32, 46, 56, 79, 130, 139]. This adds further to work on veteran identity and transition by showing how gendered ‘hero’ narratives [141] and failures to recognise women as veterans (i.e. assumptions that women are family members, or not asking women if they have served), can reduce awareness of, and referrals into, veteran-specific care [6, 7, 40, 93, 132].

### Enablers of accessing veteran care for women veterans

Enablers of access and engagement for women veterans centered on having gender-sensitive and trauma informed care [6, 7, 27–90]. Women consistently valued services that explicitly recognised their gendered experiences, including MST, caregiving roles, and discomfort in environments dominated by men [7, 36, 55, 58, 67]. Many preferred women-only spaces, women service providers, and women-only support groups [6, 7, 29, 30, 37, 42, 49, 50, 72, 73, 90]. These findings align with broader calls for gender-sensitive and trauma-informed care in civilian contexts [138], but our findings demonstrate how this approach functions specifically as a facilitator for women veterans in men-centric veteran contexts.

The centrality of empathetic, respectful service user-provider relationships and opportunities for choice (i.e. provider gender and military background, women‑only settings) also stands out [7, 46, 49, 81, 89, 99, 124, 126]. This parallels general patient-centred care literature [142–144], however, here appears closely tied to historically gendered patterns of dismissal and disbelief during military service. Where providers actively listened, acknowledged women’s service, and worked with them on treatment decisions, trust and willingness to engage with veteran services increased [7, 49, 67, 81, 99, 106, 124, 126].

Peer support is highlighted as a vital factor in encouraging women veterans to access and engage with veteran support services studies [6, 7, 28, 30–32, 38, 42, 50, 51, 56, 57, 59, 60, 71, 85, 91, 96, 113, 123, 130, 135–137]. Women described peer support and encouragement as key to navigating complex systems and overcoming anticipated stigma. This reinforces, and extends to veteran-specific settings, existing evidence that peer networks can mitigate isolation and support engagement [145, 146].

### Limitations of the evidence base

There are several limitations of the current evidence base. Most of the research in this area has been conducted in the US, within the context of the VHA. This represents a highly specialized system to provide care for veterans, which is not replicated globally. While we found parity between the US findings and the limited UK and Canadian evidence base, the transferability to other contexts is unclear.

While there was a good balance of quantitative and qualitative papers across the evidence, there was a distinct lack of papers reporting mixed-methods research designs. As there were many authors with multiple papers reported in this review, it is possible that some mixed-methods projects reported the qualitative and quantitative findings separately.

Across the evidence base, gender and sex were conceptualized inconsistently, with important implications for interpretation. Most included studies described their participants using gender terminology (e.g. women veterans), while a smaller proportion used sex-based terminology (e.g. female veterans), and nearly a quarter used sex and gender terms interchangeably. Most papers did not define eligibility of participants based on sex or gender or explain their use of this terminology. This pattern suggests limited attention to whether findings relate to biological sex, gendered social roles and norms, or both. Only a small minority of papers explicitly articulated their use of sex/gender terminology or specified eligibility for inclusion. These conceptual limitations need to be borne in mind when interpreting the gendered nature of barriers and facilitators we identify.

Finally, there is a distinct lack of an intersectional lens within the evidence base. As discussed above, sex/gender terms are used inconsistently, and without attention to the experiences of trans, non-binary, or gender diverse veterans. Only 4 of the 117 identified papers looked at women veterans from native or ethnic minority backgrounds and just five papers looked at the experiences of women veterans in rural areas. Together, these gaps mean that current knowledge is largely limited to cisgender, often urban and majority‑ethnic women veterans, and offers little insight into how overlapping intersections of marginalization shape access to and experiences of veteran‑specific services.

### Implications for future research

This review highlights several priorities for future research. First, there is a need for more studies conducted outside of the US and VHA context, including in countries where veteran care is delivered primarily through civilian systems, to understand how different service models shape women’s experiences. Second, future work should adopt clearer approaches to sex and gender, explicitly defining eligibility for participation, and inclusion of trans, non-binary and gender diverse veterans. Third, there is a shortage of research that links women’s veteran-specific service experiences to intersectional identities such as ethnicity, class, and sexuality, limiting our understanding of cumulative disadvantages. Finally, mixed-methods and longitudinal studies would help to clarify how specific barriers and experiences influence trajectories of help-seeking over time, and to evaluate the impact of interventions to promote access and engagement for women veterans.

### Implications for policy and practice

For commissioners, policymakers and service providers the findings suggest several areas for action. It is recommended that veteran-specific services should be developed and/or tailored to be gender-sensitive to women. This should include training for clinicians and support providers that provide a comprehensive understanding of women’s experiences during military service and how this may impact their support needs after service. For veteran healthcare providers, training should focus on increasing understanding of female-specific health conditions, and how these might be exacerbated by military service. Consideration must be given to ensure training and resources are culturally appropriate across different international contexts, to account for the differences in health and social care systems for veterans.

Many of the enablers of, and preferences for care reported by women across the literature, fall under the principles of providing trauma-informed care. This includes access to a safe and comfortable support environment, building a trusting relationship with service providers, supporting collaboration and shared decision-making in care, empowering service users, supporting their choices around their care, and ensuring cultural competence [147]. It is recommended that trauma-informed practice should be embedded within veteran services, to the benefit of all service users. Guidance has been developed in UK for this within the context of providing mental healthcare to women veterans [148] and could be adapted for use amongst other service providers.

Finally, our findings suggest that veteran services are perceived as being designed for men, with confusion around eligibility for services and whether they are sensitive to the needs of women. Consideration is needed in regards to the imagery and language used within service branding and documentation, to ensure that women can both see themselves represented by these services and are assured that these services can meet their needs. It is further recommended that service design and commissioning for veterans is done in collaboration and consultation with women veterans.

## Conclusions

In conclusion, this scoping review highlights the key barriers and enablers affecting women veterans’ access to veteran-specific support services. Despite differing health and social care systems internationally, the experiences of women veterans reveal common gendered barriers to engagement. Women often perceive veteran services as centered around men, leading to feelings of discomfort and exclusion, and face additional stigma in help-seeking related to derogatory perceptions of women during military service. Adverse military experiences and a lack of identification with the term ‘veteran’ further hinder their engagement with veteran-specific services. Enablers of access include competence in meeting women’s needs, positive service user-provider relationships, and peer support, underscoring the importance of trauma-informed care. Policy and practice should focus on developing gender-sensitive, culturally competent, and trauma-informed veteran services, alongside involving women veterans in service design. Addressing these gendered barriers and incorporating an intersectional lens in future research will be essential for improving the support systems for women veterans internationally.

## Supplementary Information

Supplementary Material 1.

## Data Availability

No datasets were generated or analysed during the current study.

## Abbreviations

LGBTQ+: Lesbian, Gay, Bisexual, Transgender, and Queer
NHS: National Health Service
MST: Military Sexual Trauma
PRISMA: Preferred Reporting Items for Systematic reviews and Meta-Analyses
PTSD: Post-Traumatic Stress Disorder
US: United States
UK: United Kingdom
VA: Department of Veterans’ Affairs
VHA: Veterans’ Health Administration
